# Hemostatic Parameters according to Renal Function and Time after Transplantation in Brazilian Renal Transplanted Patients

**DOI:** 10.1155/2015/472750

**Published:** 2015-07-01

**Authors:** Ana Paula Lucas Mota, Patrícia Nessralla Alpoim, Roberta Carvalho de Figueiredo, Ana Cristina Simões e Silva, Karina Braga Gomes, Luci Maria SantAna Dusse

**Affiliations:** ^1^Departamento de Analises Clínicas e Toxicológicas, Faculdade de Farmácia, Universidade Federal de Minas Gerais, 31270901 Belo Horizonte, MG, Brazil; ^2^Departamento de Ciências da Saúde, Faculdade de Farmácia, Universidade Federal de São João del-Rei, 35501296 Divinópolis, MG, Brazil; ^3^Departamento de Pediatria, Faculdade de Medicina, Universidade Federal de Minas Gerais, 30130100 Belo Horizonte, MG, Brazil

## Abstract

Kidney transplantation is the key for patients with end-stage renal disease, improving quality of life and longer survival. However, kidney transplant triggers an intense inflammatory response and alters the hemostatic system, but the pathophysiological mechanisms of these changes are not completely understood. The aim of this cross-sectional cohort study was to investigate hemostatic biomarkers in Brazilian renal transplanted patients according to renal function and time after transplantation. A total of 159 renal transplanted patients were enrolled and D-Dimer (D-Di), Thrombomodulin (TM), von Willebrand Factor (VWF), and ADAMTS13 plasma levels were assessed by ELISA. An increase of D-Di was observed in patients with higher levels of creatinine. ADAMTS13 levels were associated with creatinine plasma levels and D-Di levels with Glomerular Filtration Rate. These results suggested that D-Di and ADAMTS13 can be promising markers to estimate renal function. ADAMTS13 should be investigated throughout the posttransplant time to clarify the participation of this enzyme in glomerular filtration and acceptance or rejection of the graft in Brazilian transplanted patients.

## 1. Introduction

Kidney transplantation is the key for patients with end-stage renal disease, improving quality of life and longer survival [1–3]. Creatinine plasma levels are routinely used to define stable renal function in renal transplanted patients [4]. Clinical trials involving renal transplanted recipients usually use creatinine plasma levels and its clearance to evaluate kidney function [5, 6]. However, these markers are not accurate or sensitive to detect early changes in graft function.

Some equations based on creatinine plasma levels and other clinical parameters (age, gender, ethnicity, and serum albumin) have been developed to estimate Glomerular Filtration Rate (eGFR). The Kidney Disease Outcome Quality Initiative (K/DOQI) recommends the Modification of Diet in Renal Disease (MDRD) equation to evaluate eGFR [6]. On the other hand, there is little experience about the performance of MDRD equation in renal transplanted patients as a predictor of graft dysfunction or acute rejection [7]. Acute rejection has been associated with activation of inflammatory factors and coagulation cascade during the first three months after renal transplant. Acute rejection may result in graft loss, increased risk of chronic allograft dysfunction, and suboptimal long-term outcome [8, 9].

CKD and renal transplant are associated with activation of coagulation that favors a hypercoagulable state. Microvascular thrombosis and fibrinolytic disorders have been recognized as main cause of allograft rejection in renal transplanted patients, but the pathway through which it occurs has not been clarified yet [10–13].

Hemostatic biomarkers have been suggested to evaluate the thrombotic status and rejection risk in renal transplanted patients, mainly D-Dimer (D-Di) levels, which inform about fibrin formation and degradation [8, 10]. The aim of this study was to evaluate D-DI, TM, VWF, and ADAMTS13 plasma levels in Brazilian renal transplanted patients and investigate the association of these parameters and creatinine plasma levels, eGFR, and time (months) after transplantation.

## 2. Materials and Methods

### 2.1. Patients

A total of 159 renal transplanted patients clinically stable from two Brazilian Renal Transplant Centers (2010 to 2011) were enrolled in this study, 102 males and 57 females, with age ranging from 19 to 73 years (median = 44) and 1 to 160 months after transplantation (median = 59). All patients have received kidney from living organ donors. All patients were regularly followed up throughout the study at our outpatient health center and submitted to the same protocol of immunosuppression, which initially consisted on the combination of corticosteroid, calcineurin inhibitor (tacrolimus or ciclosporin), and mycophenolate acid according to general guidelines for renal transplantation [14, 15].

Patients with acute rejection or clinical suspicion of rejection or clinical instabilities and patients who were under hemodialysis treatment at the time of approach or had recent surgery or fractures, coagulopathies, thrombotic diseases, or acute infections or were suspected of infections on the day of blood collection were excluded from the study.

The study population was categorized into three groups according to creatinine plasma levels as C1: patients with creatinine < 1.4 mg/dL (*N* = 74); C2: patients with creatinine within 1.4–2.0 mg/dL (*N* = 60); and C3: patients with creatinine > 2.0 mg/dL (*N* = 25) or into two subgroups according to estimated Glomerular Filtration Rate (eGFR), determined by MDRD equation, eGFR < 60 mL/min/1,73 m^2^ (*N* = 48) and eGFR ≥ 60 mL/min/1,73 m^2^ (*N* = 111), or into four subgroups according to the time (months) after transplantation: T1: 1–24 months after transplant (*N* = 41); T2: 25–60 months (*N* = 40); T3: 61–120 months (*N* = 40); and T4: >120 months after transplant (*N* = 38). The major demographic and clinical features of study population are presented in Table 1.

### 2.2. Ethics

This study was approved by the Ethics Committee at Federal University of Minas Gerais/Brazil (Protocol number ETIC 387/09) and informed consent was obtained from all participants. The research protocol did not interfere with any medical recommendations or prescriptions.

### 2.3. Plasma Samples

Five mL of whole blood samples was drawn in sodium citrate 0.109 mol/L (Vacuette) and centrifuged at 1,300 g for 20 min at 4°C to obtain the plasma samples. Plasma aliquots were stored at −80°C until use for enzyme-linked immunoassay (ELISA).

### 2.4. ELISA for Hemostatic Parameters Measurements

D-Di, TM, VWF, and ADAMTS13 plasma levels were measured by specific enzyme-linked immunoassay (ELISA) kits (American Diagnostica Inc., USA), following the manufacturer's instructions.

### 2.5. Creatinine Plasma Levels and eGFR

Creatinine plasma levels were measured by specific enzymatic method, following the manufacturer's instructions (VITROS 5.1 FS). The Glomerular Filtration Rate (eGFR) was estimated by MDRD equation adapted [175 × (creatinine plasma level^−1.154^) × (age^−0.203^) × 0.742 if woman].

### 2.6. Statistical Analysis

Statistical analysis was carried out using GraphPad PRISM (version 5.0) and STATA (version 11.0) programs. Data normality was tested by Kolmogorov–Smirnov test. The interested variables D-DI, TM, and ADAMTS13 (nonparametric variables) were compared by Kruskal-Wallis test amongst groups. When differences were detected among groups, these were compared by Dunn's posttest. VWF presented normal distribution and was evaluated by ANOVA followed by “*t*” test. Initially univariate analysis was used to investigate the association between creatinine levels (dependent variable) and explanatory variables (sex, age, time after transplantation, immunosuppressive drugs therapy, D-DI, ADAMTS13, TM, and VWF levels). Creatinine levels used were <1.4 mg/dL (reference), between 1.4 and 2.0 mg/dL and > 2.0 mg/dL. Another univariate analysis was performed considering the eGFR, calculated by the MDRD formula as dependent variable (reference ≥60 mL/min/1,73 m^2^). Absolute values of independent variables (age, time after transplantation, D-DI, ADAMTS13, TM, and VWF levels) were used.

The gender variable was coded as 0: male and 1: female. The immunosuppressive drugs therapy was coded as 0: tacrolimus + mycophenolate acid + prednisone, 1: ciclosporin + mycophenolate acid + prednisone, and 3: others. Subsequently, the variables that have followed the criterion *P* < 0.20 were evaluated by multivariate logistic regression. The magnitude of the associations was measured using odds ratio (OR) and 95% confidence interval (CI) and was obtained by multiple binary and multinomial logistic regression. Correlations were determined using Spearman and Pearson rank correlation coefficients. *P* values ≤0.05 were considered statistically significant.

## 3. Results

### 3.1. Hemostatic Parameters according to Creatinine Plasma Levels and Estimated Glomerular Filtration Rate (eGFR)

**Figure 1:**
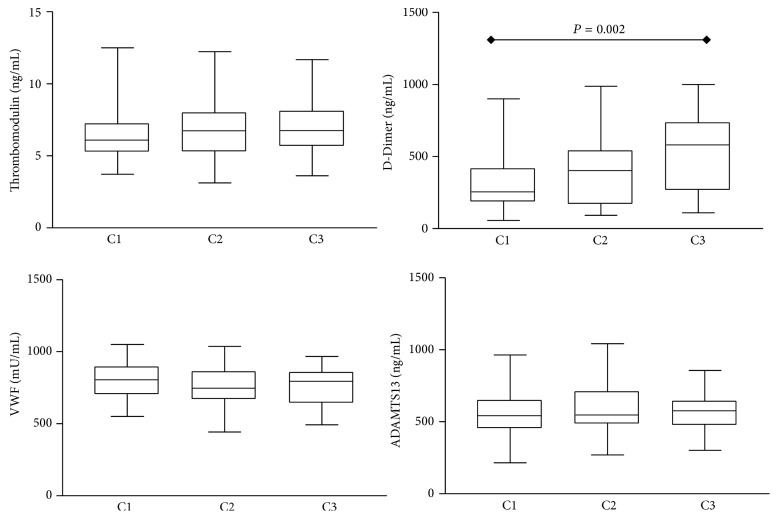
Hemostatic parameters in subgroups of renal transplanted patients according to creatinine plasma levels. Data are expressed as ng/mL (Thrombomodulin, D-Dimer (D-DI), and ADAMTS13) and presented as median + interquartile range or mU/mL (von Willebrand Factor (VWF)) and presented as mean ± standard deviation. The subgroups are C1 (creatinine < 1.4 mg/dL), C2 (creatinine 1.4–2.0 mg/dL), and C3 (creatinine > 2.0 mg/dL). Significant differences at *P* < 0.05 are highlighted by connecting lines.

**Figure 2:**
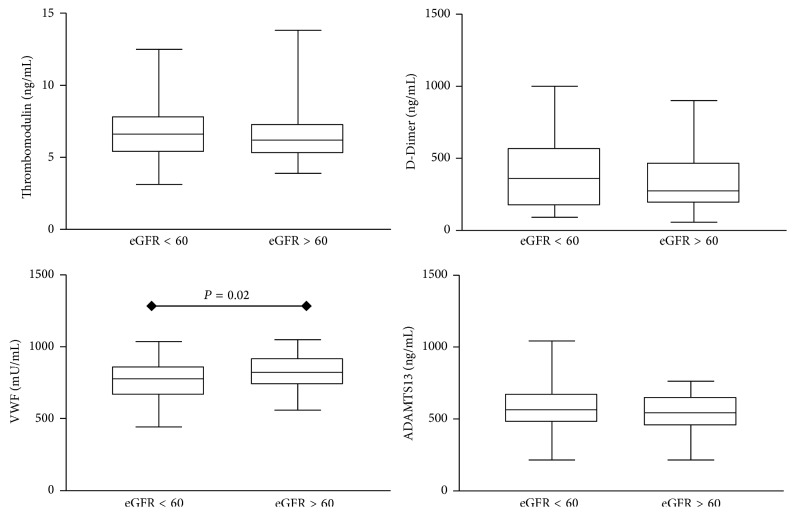
Plasma levels of hemostatic parameters in subgroups of renal transplanted patients according to estimated Glomerular Filtration Rate (eGFR). Data are expressed as ng/mL (Thrombomodulin, D-Dimer (D-DI), and ADAMTS13) and presented as median + interquartile range or mU/mL (von Willebrand Factor (VWF)) and presented as mean ± standard deviation. The subgroups are eGFR < 60 and eGFR > 60 mL/min/1.73 m^2^. Significant differences at *P* < 0.05 are highlighted by connecting lines.

The hemostatic parameters D-DI, TM, VWF, and ADAMTS13 were available in 159 renal transplanted patients according to creatinine plasma levels (subgroups C1, C2, and C3) and eGFR (subgroups eGFR < 60 and eGFR ≥ 60). It is important to mention that this is the first time that these four biomarkers are assessed in Brazilian renal transplanted patients at the same time. Comparisons of hemostatic parameters observed at each subgroup are shown in Figures 1 and 2. Our findings demonstrated higher levels of D-DI (*P* = 0.002) in subgroup C3 (509 ng/mL) with creatinine >2.0 mg/dL as compared to C1 (336 ng/mL) with creatinine < 1.4 mg/dL (Figure 1). Higher levels of VWF (*P* = 0.020) were observed in subgroup with eGFR ≥ 60 (813.5 mU/mL) as compared to eGFR < 60 (778.5 mU/mL) (Figure 2). No significant differences were observed for the other biomarkers evaluated. There were positive correlations between creatinine and TM (*P* = 0.010) and creatinine and D-DI (*P* < 0.001). No significant correlations were observed between other biomarkers (VWF and ADAMTS13) and creatinine or eGFR.

### 3.2. Hemostatic Parameters according to Time after Transplantation

**Figure 3:**
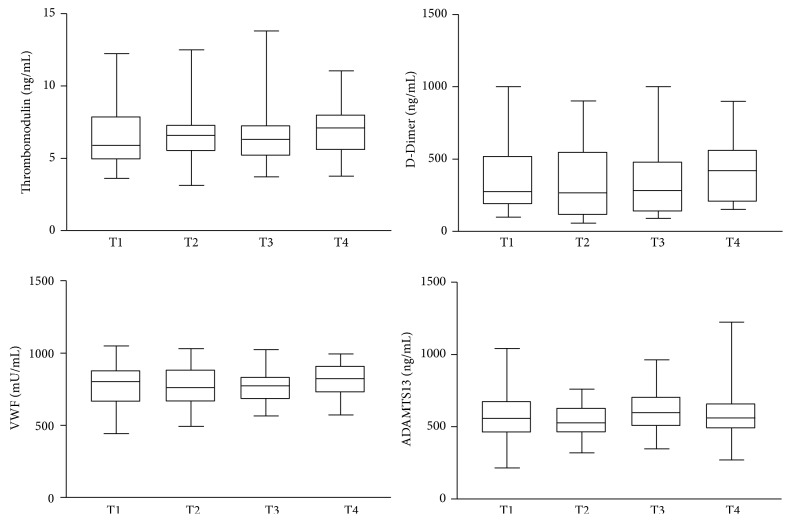
Hemostatic parameters plasma levels in subgroups of renal transplanted patients according to time after transplant. Data are expressed as ng/mL (Thrombomodulin, D-Dimer (D-DI), and ADAMTS13) and presented as median + interquartile range or mU/mL (von Willebrand Factor (VWF)) and presented as mean ± standard deviation. The subgroups are T1 (1–24 months), T2 (25–60 months), T3 (61–120 months), and T4 (>120 months after transplant). No significant differences were observed (*P* < 0.05).

D-DI, TM, VWF, and ADAMTS13 levels were not different comparing the four subgroups according to time after transplantation (T1, T2, T3, and T4), as shown in Figure 3.

### 3.3. Univariate and Multivariate Analysis in Function of Creatinine Plasma Levels

An additional strategy of data analysis was used to each hemostatic parameter seeking associations with creatinine plasma levels. These analyses are shown in Tables 2(a) and 2(b).

Preliminary analysis revealed significant association between creatinine plasma levels (>2.0 mg/dL) and two hemostatic parameters: D-DI (OR = 1.01; *P* = 0.01) and TM (OR = 1.19; *P* = 0.03). The VWF and ADAMTS13 presented *P* < 0.20 and were inserted into the subsequent multivariate analysis (Table 2(a)).

In multivariate analysis, we observed an association between ADAMTS13 and creatinine plasma levels (OR = 1.01; *P* = 0.05), among D-DI and creatinine (OR = 1.00; *P* = 0.03) and TM with creatinine levels (OR = 1.20; *P* = 0.03) (Table 2(b)).

### 3.4. Univariate and Multivariate Analysis in Function of Estimated Glomerular Filtration Rate (eGFR)

These analyses are shown in Tables 3(a) and 3(b). Considering the two categories of the response variable (eGFR < and ≥ 60 mL/min/1,73 m^2^), the univariate logistic regression showed significant association between eGFR and D-DI (OR = 1.00; *P* = 0.04) as showed in Table 3(a). Only TM and ADAMTS13 presented *P* ≤ 0.20 and were inserted into the subsequent multivariate analysis.

Our subsequent multivariate logistic regression analysis revealed that D-DI (OR = 1.00; *P* = 0.04) was independently correlated with eGFR < 60 mL/min/1,73 m^2^ (Table 3(b)). No significant differences were observed for the other hemostatic biomarkers.

## 4. Discussion

Prediction of early and late allograft function is central in kidney transplantation, as this may permit tailoring of medical management to maximize organ recovery [1, 2]. Serial measurements of serum creatinine and renal biopsy have not been able to detect early alterations of graft function [16]. Unfortunately, serum creatinine measurement is not a good indicator of acute renal function alterations [1, 2]. The technology employed for biopsy assessment and the resulting diagnostic classification did however not always keep pace with the rapidly evolving knowledge about the mechanisms of rejection [2]. Therefore, new biomarkers for early predicting renal graft dysfunctions are clearly needed [4, 8, 9]. In this regard, it is well known that the activation of blood coagulation or suppression of fibrinolysis plays a role in the progression of atherosclerosis in renal transplanted patients and it seems to be the major cause of mortality after transplant. Despite many years of intensive research, the cause of hemostatic changes after renal transplantation is not fully understood [7, 10].

The patients of this study were regularly followed up throughout at our outpatient health center and it is important to mention that, during the follow-up, calcineurin inhibitor (tacrolimus or ciclosporin) was replaced by Sirolimus/Rapamycin in 8 patients (5.03%) and corticosteroid therapy (prednisone) was removed due to adverse effects in 9 patients (5.66%). Other immunosuppression modifications were made as the exclusion of mycophenolate acid, tacrolimus, or ciclosporin (2 patients/1.25%) and the replacement of mycophenolate acid by Everolimus in 2 patients (1.25%). These alterations did not interfere with the logistic regression analysis. Indeed, no differences were observed for the evaluated hemostatic parameters according to immunosuppressive drugs used.

The hemostasis/fibrinolysis evaluation in renal transplanted patients in this study included the determination of four biomarkers: D-DI, TM, VWF, and ADAMTS13 levels. Our research group have previously studied these hemostatic markers in the same group of patients before renal transplantation and showed that the imbalance between ADAMTS13 and VWF levels may contribute to the hypercoagulability state [17, 18]. In the present, our data showed higher D-DI plasma levels in subgroup C3 (creatinine > 2.0 mg/dL) as compared to C1 (creatinine < 1.4 mg/dL), which suggest that impaired glomerular filtration influences D-DI clearance and may favor a thrombotic or hypofibrinolytic state. In fact, other studies also reported an increase in D-DI plasma levels in a short-term after transplantation [5, 11]. After surgery, in the immediate posttransplant, it is really expected to increase D-DI plasma levels, but it decreases with regression of creatinine plasma levels sometime after transplantation and stable graft function.

Most studies evaluated the hemostatic markers only short-term after renal transplantation [8, 11] and showed that higher D-DI levels are expected immediately after surgery, but this hemostatic change could be corrected after a successful transplant. There are few studies associating renal function, creatinine, and D-DI plasma levels, as well as other hemostatic biomarkers, in long-term posttransplant [12, 13, 19–25]. Some previous studies demonstrated endothelial injury, enhanced coagulation, and fibrinolytic system impairment, in long-term posttransplant [5, 10, 11, 26–33]. In our study, no significant difference was found for other biomarkers (TM, VWF, and ADAMTS13 levels) according to time after transplantation. Nevertheless it is believed that there is influence of time after transplantation in the graft function.

On the other hand, high D-DI plasma levels can also be consequent to the reduction of its urinary clearance and lower eGFR. D-Di has high molecular weight and could not be lost in the urine in intact form. Thus, detection of D-Di fragments in urine can be used as an auxiliary method of reversibility of acute rejection or chronic allograft nephropathy in renal transplant recipients [12, 13]. Moreover, D-Di is a classic marker of fibrin degradation [10, 11, 20] and further studies are needed to clarify its role in renal transplanted patients.

Changes on endothelial function precede the development of atherosclerosis and can also contribute to lesion development and later clinical complications. TM, an endothelial lesion marker, is higher in thrombotic disorders as it may occur in renal transplant [20, 21, 34–36]. In our study, no significant differences were found in TM levels according to creatinine levels, but the subgroups medians values (C1 = 6.12; C2 = 6.76; and C3 = 7.02 ng/mL) were above the reference values (4.0 to 5.35 ng/mL). In agreement with our results, several researchers had showed TM increasing after renal transplantation [7, 10, 22–25]. No significant differences were found in TM levels according to eGFR (Figure 2), but patients with eGFR < 60 mL/min/1.73 m^2^ had slightly higher levels comparing to patients with eGFR ≥ 60. Previous study has shown an increase in TM levels in patients with eGFR < 60 and acute kidney injury [12]. It is known that endothelium contributes to normal hemostasis and the control of excess blood clotting. TM has an essential role in the protein C pathway, one natural anticoagulant. Smooth muscle cells, platelets, monocytes, and cardiomyocytes also express TM [10, 26, 33–36]. It can justify the similar TM levels in all transplanted patients, since the expression of TM in other tissues may have hidden its relevance in the kidney. Moreover, we systemically evaluate the expected intrarenal response. TM was also detected in tumor cells, suggesting that its biological function is not restricted to natural anticoagulation [26]. Therefore, TM could be a promising marker of endothelial damage in kidney transplanted patients. TM plasma levels above the reference range could also be explained by use of cyclosporine. Increased levels of TM soluble in cultured cells after addition of cyclosporine in the culture were shown [27].

VWF levels according to creatinine plasma levels (C1 = 796.0; C2 = 757.9; C3 = 770.2 mU/mL) were within the reference range (683 to 1012 mU/mL). No difference among subgroups was found, but when VWF was assessed in function of eGFR there are higher levels of this biomarker in patients with eGFR ≥ 60 mL/min/1.73 m^2^ (813.5 mU/mL) as compared to patients with eGFR < 60 (773.4 mU/mL). One of the main functions of VWF is to induce platelet thrombus at sites of vascular injury and high-shear stress. This function is dependent on the size of the VWF multimers and function of ADAMTS13 [28, 29, 37–39]. A possible explanation of higher VWF levels in patients with eGFR ≥ 60 could be lower ADAMTS13 levels, also detected in these patients.

Previous study reported that treatment with corticosteroids or mycophenolate acid reduced VWF levels in patients with lupus nephritis, suggesting that these immunosuppressive drugs improve endothelial function. In our study all patients used one of these drugs, which may have contributed to maintaining the average VWF within the reference range [29, 37–39]. Contrary to the results obtained in the present study, increased levels of VWF in renal transplanted patients with stable function, associated with worsening renal function, were previously reported [11, 22, 23, 25].

ADAMTS13 median did not differ in the three subgroups according to creatinine levels (C1 = 544.3; C2 = 547.6; and C3 = 576.3 ng/mL) and in the two subgroups according to eGFR (eGFR < 60 = 561.8; eGFR ≥ 60 = 544.3), but it was below the reference values (630 to 860 ng/mL). ADAMTS13 mRNA has been detected in a variety of tissues, including the kidney. ADAMTS13 regulates the size and thereby the activity of VWF multimers through rapid cleavage upon their release from endothelial cells. Decrease of ADAMTS13 and deficient VWF cleavage allows ultralarge VWF (ULVWF) to accumulate in the circulation and contribute to thrombus formation [28, 37, 39]. These investigators showed that ADAMTS13 was detected in the urine of patients with tubular damage, but not in individuals with healthy renal function, because it is a large protein that would not be expected to be filtered [28, 30, 37, 39]. This phenomenon is important under conditions of blood flow associated with high-shear stress as into the kidney microcirculation, especially after renal transplant [29, 30].

Univariate analysis of multinomial regression revealed a significant association between creatinine levels (>2 mg/dL) and hemostatic parameters (D-Di, TM). In subsequent multivariate analysis, D-Di, TM, and ADAMTS13 remained positively associated with creatinine plasma levels. In fact, D-Di levels were also higher in subgroup C3 and were associated with eGFR decline. In this study, despite no differences, ADAMTS13 levels were higher in subgroup C3 as compared to C2 or C1. In fact, in patients with high creatinine levels, the renal filtration capacity could be compromised, which could lead to lower ADAMTS13 clearance.

As previously mentioned, higher levels of TM were found in patients with eGFR < 60 mL/min/1,73 m^2^ and higher creatinine levels [12, 29]. Other previous studies have reported that endothelial damage is more pronounced in patients transplanted with lower eGFR [11, 22, 23].

Assessment of renal function is essential for kidney transplant management. It has been a challenge to prevent early graft loss since the defective renal function is not detected until creatinine plasma levels have risen above baseline. Creatinine plasma levels are affected by many factors, such as muscle mass, gender, diet, liver function, medications, and time after transplant [5–7, 40, 41]. Considering the limitations of creatinine plasma levels to assess renal function, the eGFR was used for univariate and multivariate analysis in this study too. In summary, hemostatic parameters (especially DD, TM, and ADAMTS13) were associated with creatinine plasma levels and graft function. Despite the fact that the strategy to analyze the relation between biomarkers and creatinine clearance had already been used in other studies [42], the results obtained should be interpreted with caution. Therefore, our findings did not allow us to define which hemostatic marker should be measured during the follow-up of renal transplanted patients. In addition, we were not able to clearly establish which marker could predict late renal function. On the other hand, despite the low number of patients, the present study indicates that the molecules D-Di and ADAMTS13 could be related, in a different way, with late renal function after transplantation. Importantly, preliminary results showed that the high D-Di correlated with subsequent worsening of kidney function in five patients and one patient died due to thrombosis and acute renal artery occlusion. This suggests that prospective studies should be performed to assess the role of D-Di on this context.

Further studies with high number of transplanted subjects are obviously necessary to investigate the role of these molecules measurements as a tool in the follow-up of renal transplanted patients.

## 5. Conclusions

Our data showed that D-Di levels were higher according to creatinine plasma levels and there was a tendency to elevate in subgroups (C3 > C2 > C1), which also explains the association with lower eGFR. Therefore, the role of hemostatic markers, in particular, D-Di, TM, and ADAMTS13, should be further explored in future studies.

Taken together, our data suggest that D-Di was the promising marker for estimating renal function. Moreover, the levels of ADAMTS13 should be investigated throughout the posttransplant time to clarify the participation of this enzyme in glomerular filtration and graft rejection.

## Figures and Tables

**Table 1:** Demographic and biochemical data of patients.

Characteristic	Value
Age (yr)	44 (19–73)
Sex	
Male	102 (64.1%)
Female	57 (35.9%)
BMI (Kg/m^2^)	24.8 (17.6–34.7)
Creatinine levels (mg/dL)	1.38 (0.59–3.62)
eGFR (mL/min/1.73 m^2^)	59.17 (18.24–103.97)
Cholesterol (mg/dL)	187.5 (111.0–341.0)
Triglycerides (mg/dL)	143.5 (50.0–552.0)
HDL cholesterol (mg/dL)	31.7 (23–68.2)
LDL cholesterol (mg/dL)	120.1 (78.0–219.2)
Time after transplant (months)	59 (1–160)
Time of dialysis (months)	15 (2–30)
Cause of renal disease	
Glomerulonephritis	27 (17.0%)
Hypertension	38 (23.9%)
Diabetes	18 (11.3%)
Others	10 (6.3%)
Unknown	66 (41.5%)

Values are presented as median (range) or number (%). BMI: body mass index; eGFR: estimated glomerular filtration rate.

**(a):** 

Variables	Creatinine 1.4–2.0 mg/dL			Creatinine > 2.0 mg/dL		
	OR	OR (95% CI)	*P* value	OR	OR (95% CI)	*P* value
DDI	1.00	0.99; 1.00	0.09	1.01	1.01; 1.03	0.01^*∗*^
TM	1.12	0.97; 1.29	0.12	1.19	1.02; 1.39	0.03^*∗*^
VWF	0.99	0.99; 1.00	0.17	0.99	0.99; 1.00	0.45
ADAMTS13	1.00	0.99; 1.00	0.17	1.01	0.99; 1.00	0.32

^*∗*^Significant (*P* ≤ 0.05).

**(b):** 

Variables	Creatinine 1.4–2.0 mg/dL			Creatinine > 2.0 mg/dL		
	OR	OR (95% CI)	*P* value	OR	OR (95% CI)	*P* value
DDI	1.00	0.99; 1.00	0.17	1.00	0.99; 1.00	0.03^*∗*^
TM	1.12	0.97; 1.30	0.14	1.20	1.02; 1.42	0.03^*∗*^
VWF	0.99	0.99; 1.00	0.16	0.99	0.99; 1.00	0.64
ADAMTS13	1.01	0.99; 1.00	0.09	1.01	0.99; 1.00	0.05^*∗*^

^*∗*^Significant (*P* ≤ 0.05).

**(a):** 

Variables	eGFR < 60 mL/min/1.73 m^2^		
	OR	CI	*P* value
DDI	1.00	1.00; 1.01	0.04^*∗*^
TM	1.13	0.96; 1.32	0.13
VWF	0.99	0.99; 1.00	0.39
ADAMTS13	1.00	0.99; 1.00	0.20

^*∗*^Significant (*P* ≤ 0.05). Variables with *P* < 0.20 were included in the multivariable analysis.

**(b):** 

Variables	eGFR < 60 mL/min/1.73 m^2^		
	OR	CI	*P* value
DDI	1.00	1.00; 1.01	0.04^*∗*^
TM	1.18	0.88; 1.59	0.27
ADAMTS13	1.00	0.99; 1.00	0.12

^*∗*^Significant (*P* ≤ 0.05).
